# Supplementary material for: Phase separation of a microtubule plus-end tracking protein into a fluid fractal network
Source: Nat Commun. 2025 Jan 30;16:1165. doi: 10.1038/s41467-025-56468-8 (PMC11782662; doi:10.1038/s41467-025-56468-8)
Supplement: Supplementary file 1 — Supplementary Information [file 41467_2025_56468_MOESM1_ESM.pdf]

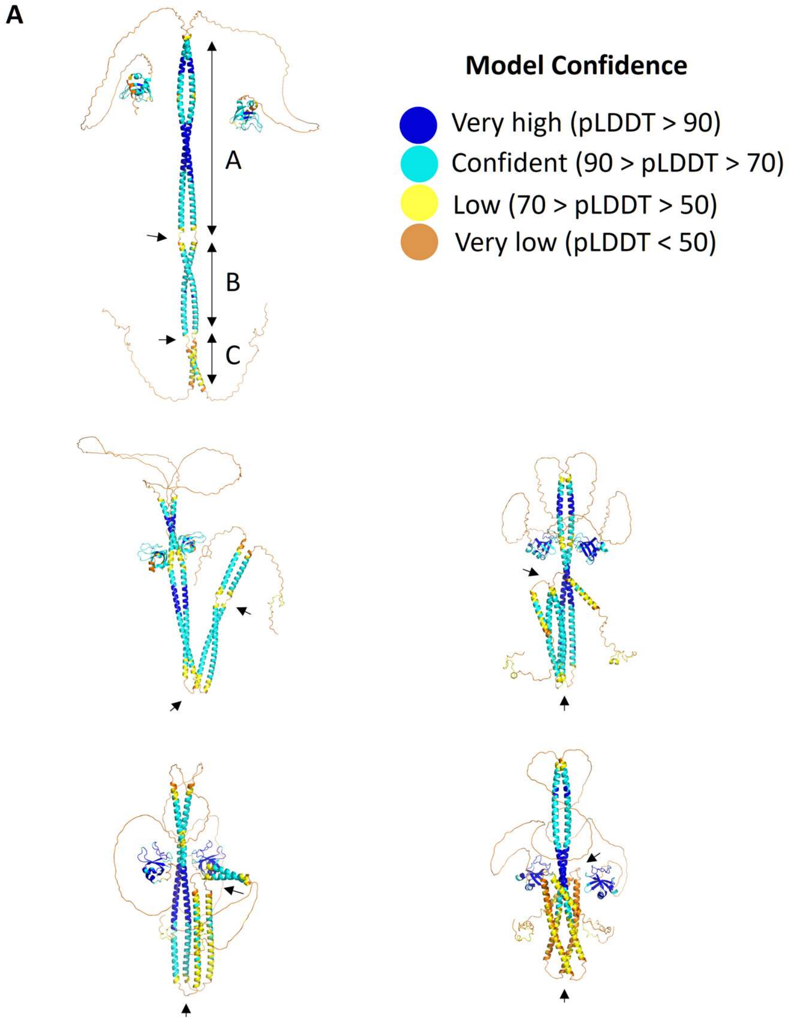

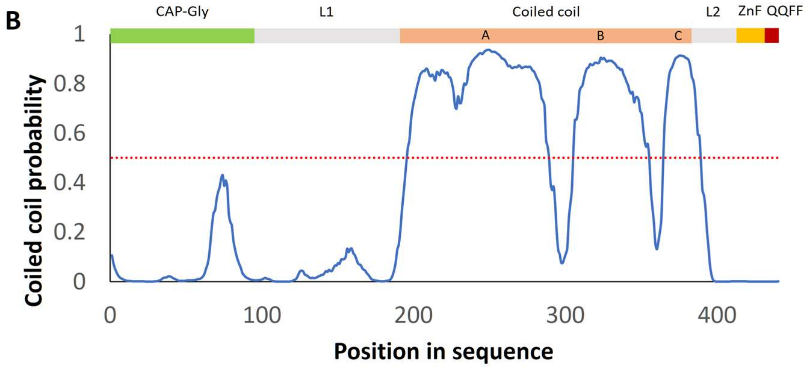

Supplementary Figure 1. Computational predictions of the Bik1 structure. A) Different possible structures of the Bik1 dimer predicted by AlphaFold.[1] Structures are color-coded according to the pLDDT score: regions with a high pLDDT score (>90) are expected to be modeled with high accuracy, while regions with a low pLDDT score (<70) were modeled with low confidence. Black single arrows indicate predicted discontinuities in the coiled-coil domain; double black arrows indicate the three coiled-coil segments A, B, and C. B) Sequence-based coiled-coil prediction of Bik1 by DeepCoil.[2] The blue line represents the probability of coiled-coil formation per residue; the red dashed line indicates the 50% probability threshold. ZnF, zinc finger domain; EEY/F-like motif Gln-Gln-Phe-Phe. The amino acid sequence used for the predictions are reported in the Source Data file.

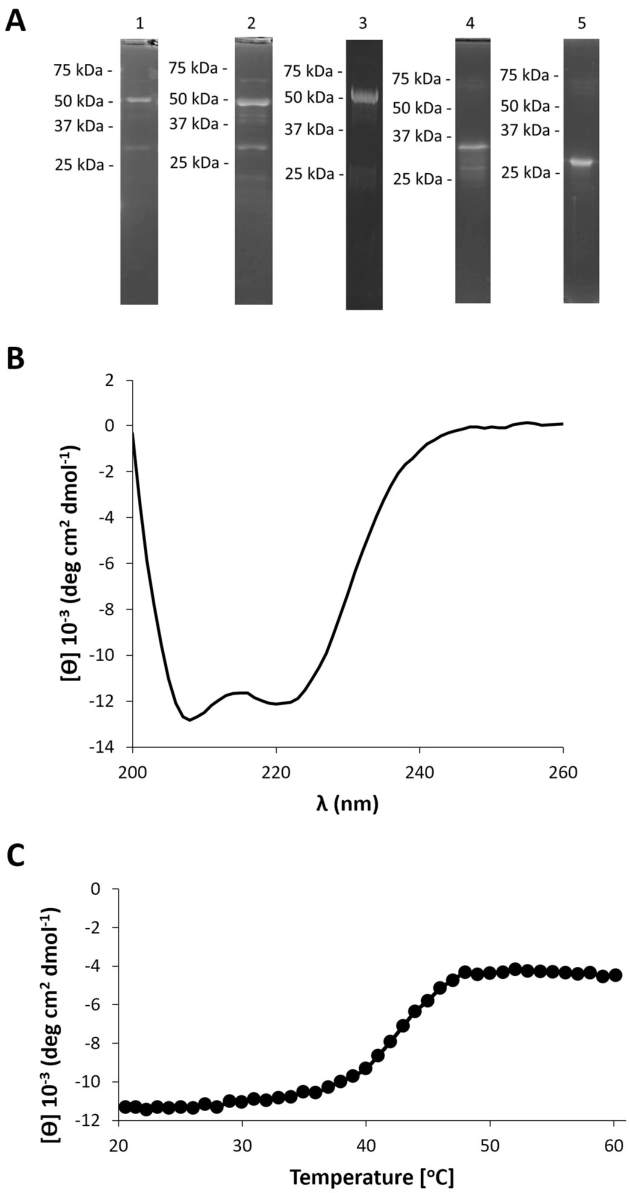

Supplementary Figure 2. Quality assessment of Bik1 samples. A) Coomassie Blue-stained SDS-PAGE analysis of Bik1 protein variants used in this study: 1, Bik1 FL; 2, Bik1 FL cleaved (N-terminal His-tag removed); 3, Bik1 ΔQQFF; 4, Bik1 ΔN; 5, Bik1 ΔNC. All samples apart from Bik1 FL cleaved contained an N-terminal His-tag. Spectrum (B) and thermal unfolding profile (C) recorded by CD at 222 nm for Bik1 FL in high-salt buffer conditions (20 mM Tris-HCl, pH 7.4, supplemented with 500 mM NaCl and 10% glycerol). Source data for all panels are provided in the Source Data file.

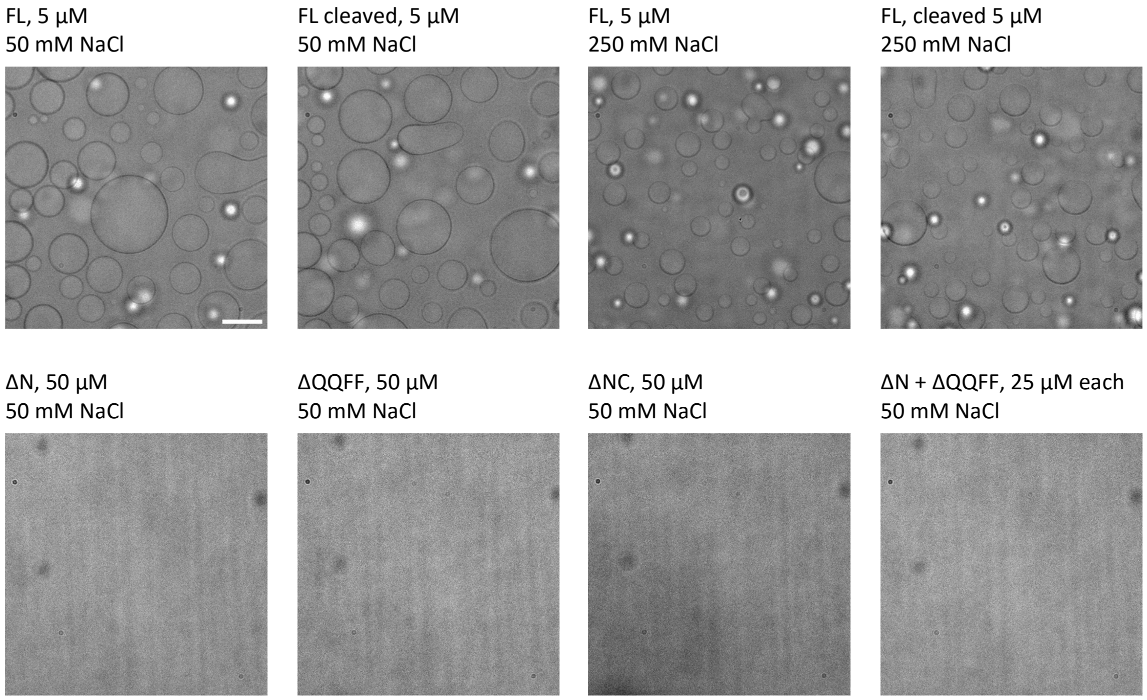

Supplementary Figure 3. Phase separation of Bik1 variants. Bright-field microscopy images of Bik1 FL and its truncated variants (10 mM HEPES, pH 7.5, supplemented with 50 or 250 mM NaCl, 1 mM DTT, and 10% glycerol). Bik1 FL cleaved, full-length protein with N-terminal His tag removed. Scale bar, 10 µm.

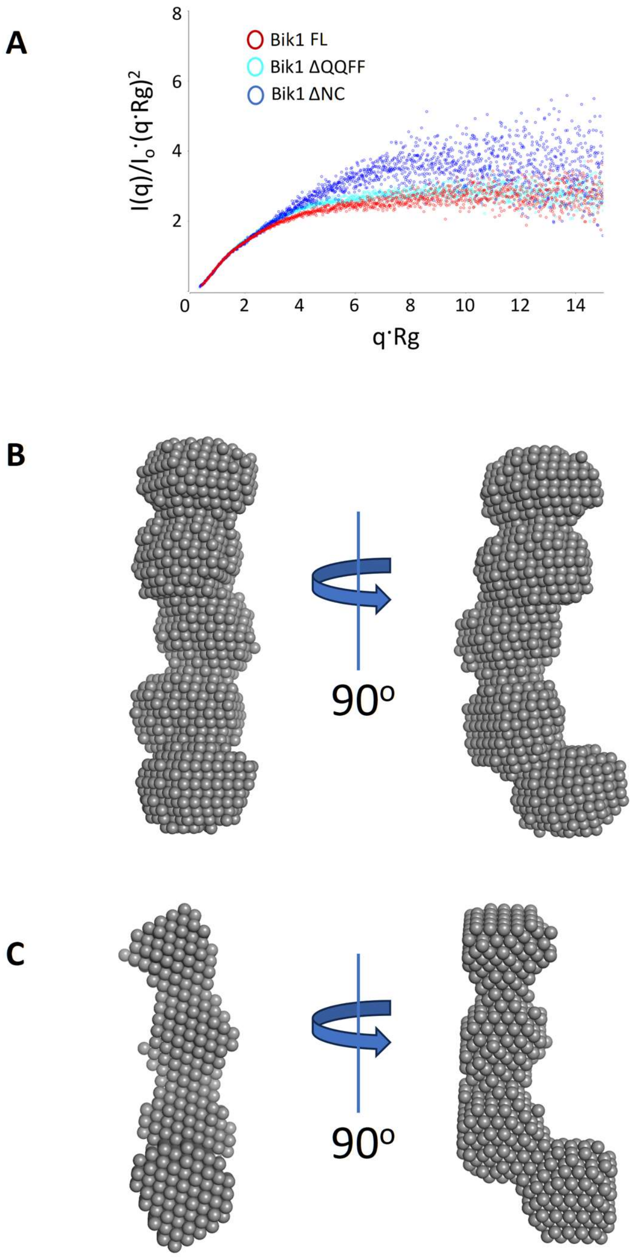

Supplementary Figure 4. SEC-SAXS of Bik1 variants. A) Dimensionless Kratky plots obtained for Bik1 FL, Bik1 ΔQQFF, and Bik1 ΔNC. B and C) DAMMIF generated molecular envelopes (beads model) of Bik1 ΔQQFF (B) and Bik1 ΔNC (C) calculated by averaging 19 models (out of a total of 20) resulting in NSD values of 0.71 ± 0.05 and 0.61 ± 0.04, respectively. The resolution of ensembles is 65 ± 5 Å for Bik1 ΔQQFF and 43 ± 3 Å for Bik1 ΔNC.

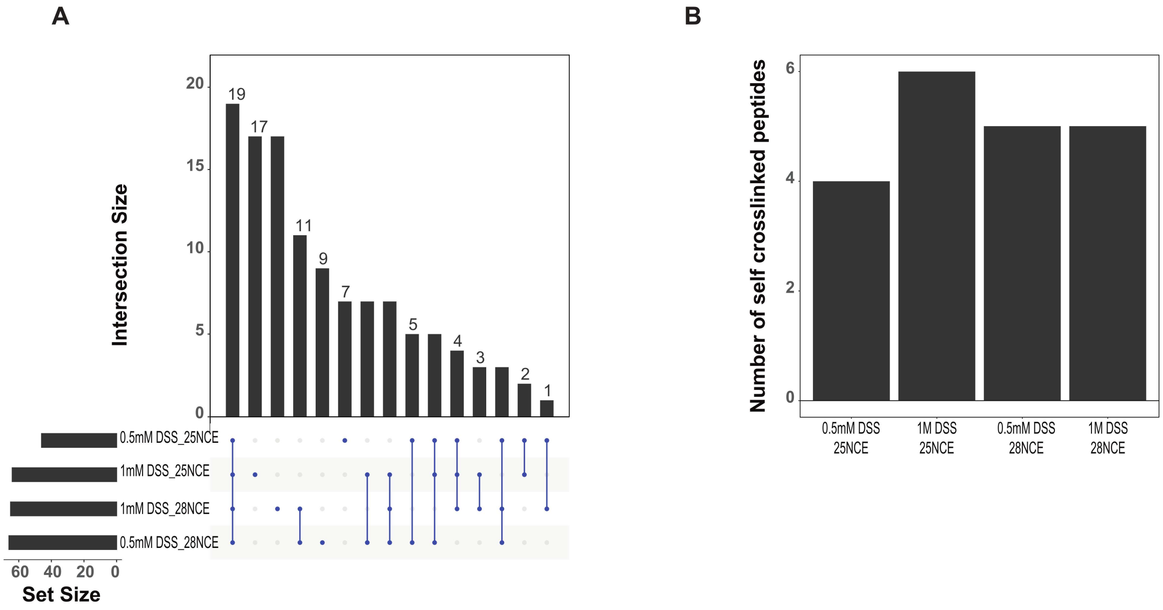

Supplementary Figure 5. Analysis of different crosslinked Bik1 FL samples. A) Upset plot of identified crosslinked peptides using different concentrations of DSS and distinct acquisition method based on varying collision energy. B) Identification of self-linked peptides of different experimental conditions (different concentrations of DSS reagent and collision energy). Data in both panels represent the results of single experiments. NCE, normalized collision energy for MS fragmentation. Source data for both panels are provided in the Source Data file.

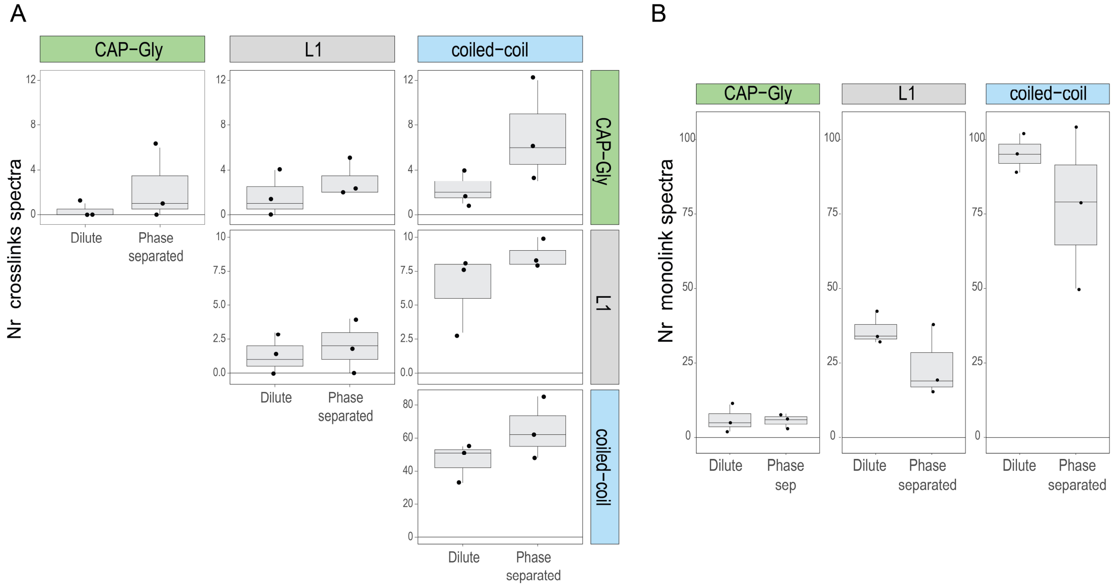

Supplementary Figure 6. Number of identified spectra associated to Bik1 domains. A) Number of identified spectra for intra crosslinked DSS peptides associated to the following Bik1 domains and regions: CAP-Gly (residues 1-80), disordered L1 (residues 81-188) and coiled coil (residues 189-389). B) Number of identified spectra for DSS monolink peptides associated to the Bik1 domain and regions described in panel A. Each dot represents the result of an independent experiment (*N*=3) with the box plot boundaries indicating the quantiles Q1 (25%) and Q3 (75%), the lower and upper whiskers are defined by Q1 − 1.5 IQR and Q3 + 1.5 IQR, and the median value denoted by a line across the box. Source data for both panels are provided in the Source Data file.

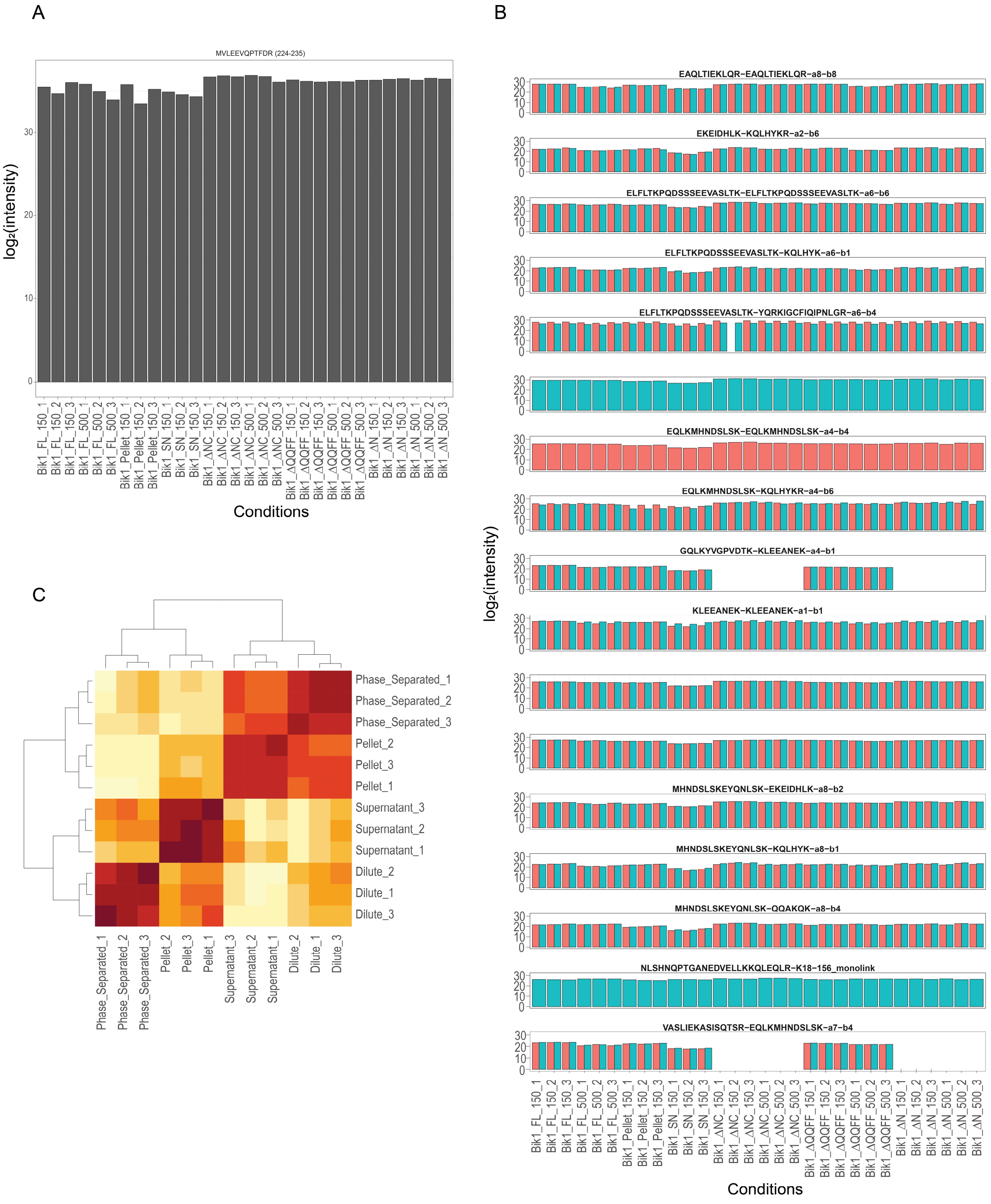
A
B
C
MVLEEVQPTFDR (224-235)
log2(intensity)
Conditions
EAQLTIEKLQR–EAQLTIEKLQR–a8–b8
EKEIDHLK–KQLHYKR–a2–b6
ELFLTKPQDSSSEEVASLTK–ELFLTKPQDSSSEEVASLTK–a6–b6
ELFLTKPQDSSSEEVASLTK–KQLHYK–a6–b1
ELFLTKPQDSSSEEVASLTK–YQRKIGCFIQIPNLGR–a6–b4
EQLKMHNDSLSK–EQLKMHNDSLSK–a4–b4
EQLKMHNDSLSK–KQLHYKR–a4–b6
GQLKYVGPVDTK–KLEEANEK–a4–b1
KLEEANEK–KLEEANEK–a1–b1
MHNDSLSKEYQNLSK–EKEIDHLK–a8–b2
MHNDSLSKEYQNLSK–KQLHYK–a8–b1
MHNDSLSKEYQNLSK–QQAKQK–a8–b4
NLSHNQPTGANEDVELLKKQLEQLR–K18–156_monolink
VASLIEKASISQTSR–EQLKMHNDSLSK–a7–b4
Bik1_FL_150_1
Bik1_FL_150_2
Bik1_FL_150_3
Bik1_FL_500_1
Bik1_FL_500_2
Bik1_FL_500_3
Bik1_Pellet_150_1
Bik1_Pellet_150_2
Bik1_Pellet_150_3
Bik1_SN_150_1
Bik1_SN_150_2
Bik1_SN_150_3
Bik1_ΔNC_150_1
Bik1_ΔNC_150_2
Bik1_ΔNC_150_3
Bik1_ΔNC_500_1
Bik1_ΔNC_500_2
Bik1_ΔNC_500_3
Bik1_ΔQQFF_150_1
Bik1_ΔQQFF_150_2
Bik1_ΔQQFF_150_3
Bik1_ΔQQFF_500_1
Bik1_ΔQQFF_500_2
Bik1_ΔQQFF_500_3
Bik1_ΔN_150_1
Bik1_ΔN_150_2
Bik1_ΔN_150_3
Bik1_ΔN_500_1
Bik1_ΔN_500_2
Bik1_ΔN_500_3
Phase_Separated_1
Phase_Separated_2
Phase_Separated_3
Pellet_2
Pellet_3
Pellet_1
Supernatant_3
Supernatant_2
Supernatant_1
Dilute_2
Dilute_1
Dilute_3

D

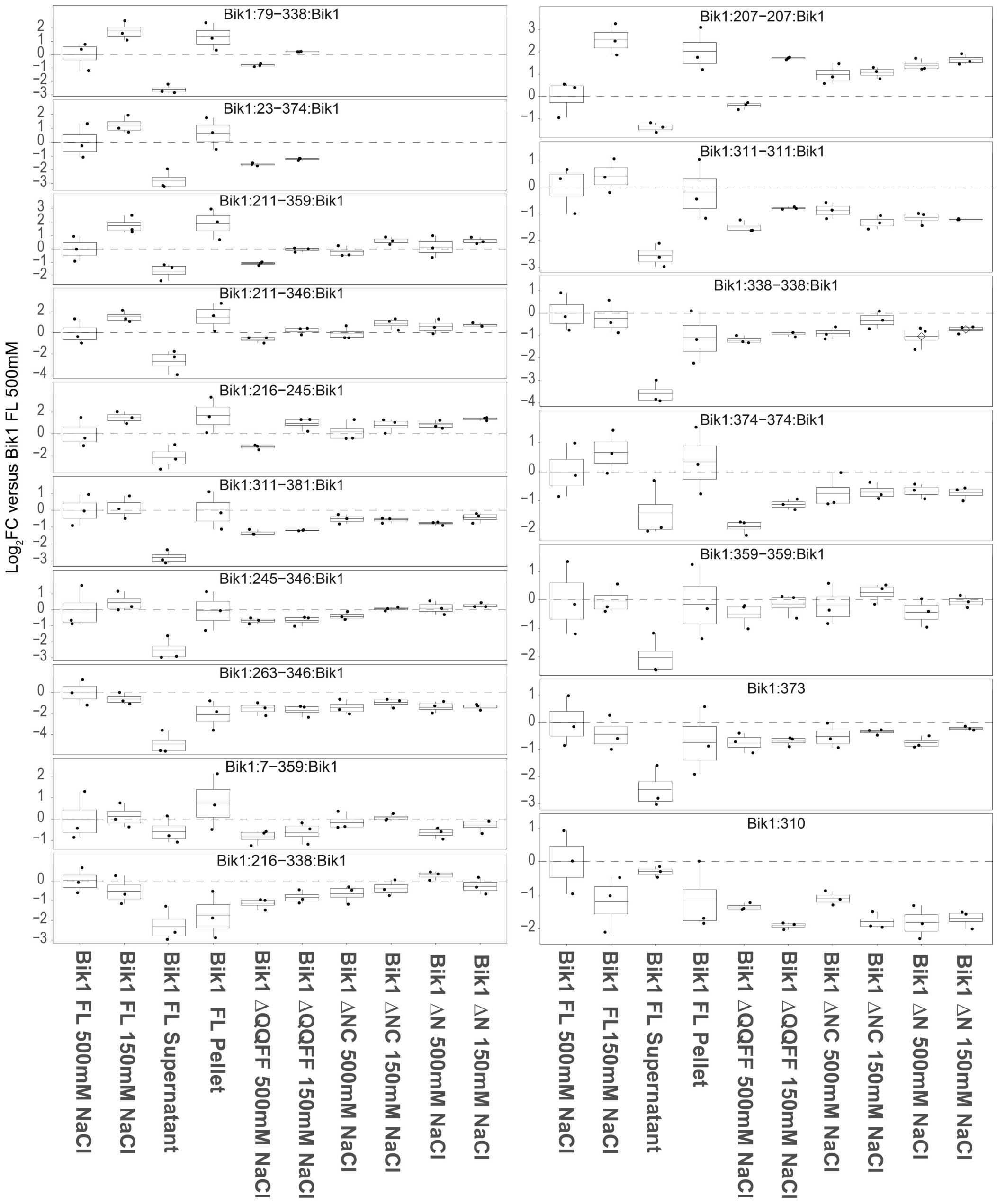

Supplementary Figure 7. Quality control of targeted XL-MS analysis. A) Intensity abundance of Bik1 FL (expressed as log2) for all acquired runs. The reported intensity is derived from the intensity of peptide MVLEEVQPTFDR (residues 224-235 of Bik1). Bik1 abundance serves as a normalization factor for crosslink intensity to control protein recovery levels. B) Intensity abundance (expressed as log2) for crosslinked peptides monitored across all acquired runs. Cyan and red bars indicate the abundance of heavy and light crosslinked peptides, respectively. C) Unsupervised hierarchical cluster analysis based in Pearson correlation for conformospecific crosslink peptides across the tested conditions. D) Boxplot showing the abundance of all measured crosslinked peptides normalized for Bik1 abundance at high (500 mM) and low (150) NaCl concentration. The following Bik1 variants have been screened in three independent experiments: Bik1 FL, Bik1 ΔN, Bik1ΔNC, and Bik1ΔQQFF. Each dot represents the results of an independent experiment, with the box plot boundaries indicating the quantiles Q1 (25%) and Q3 (75%), the lower and upper whiskers are defined by Q1 − 1.5 IQR and Q3 + 1.5 IQR, and the average value denoted by a line across the box. Data are normalized for the average intensities at high salt concentration of the Bik1 FL condition. Source data for all panels are provided in the Source Data file.

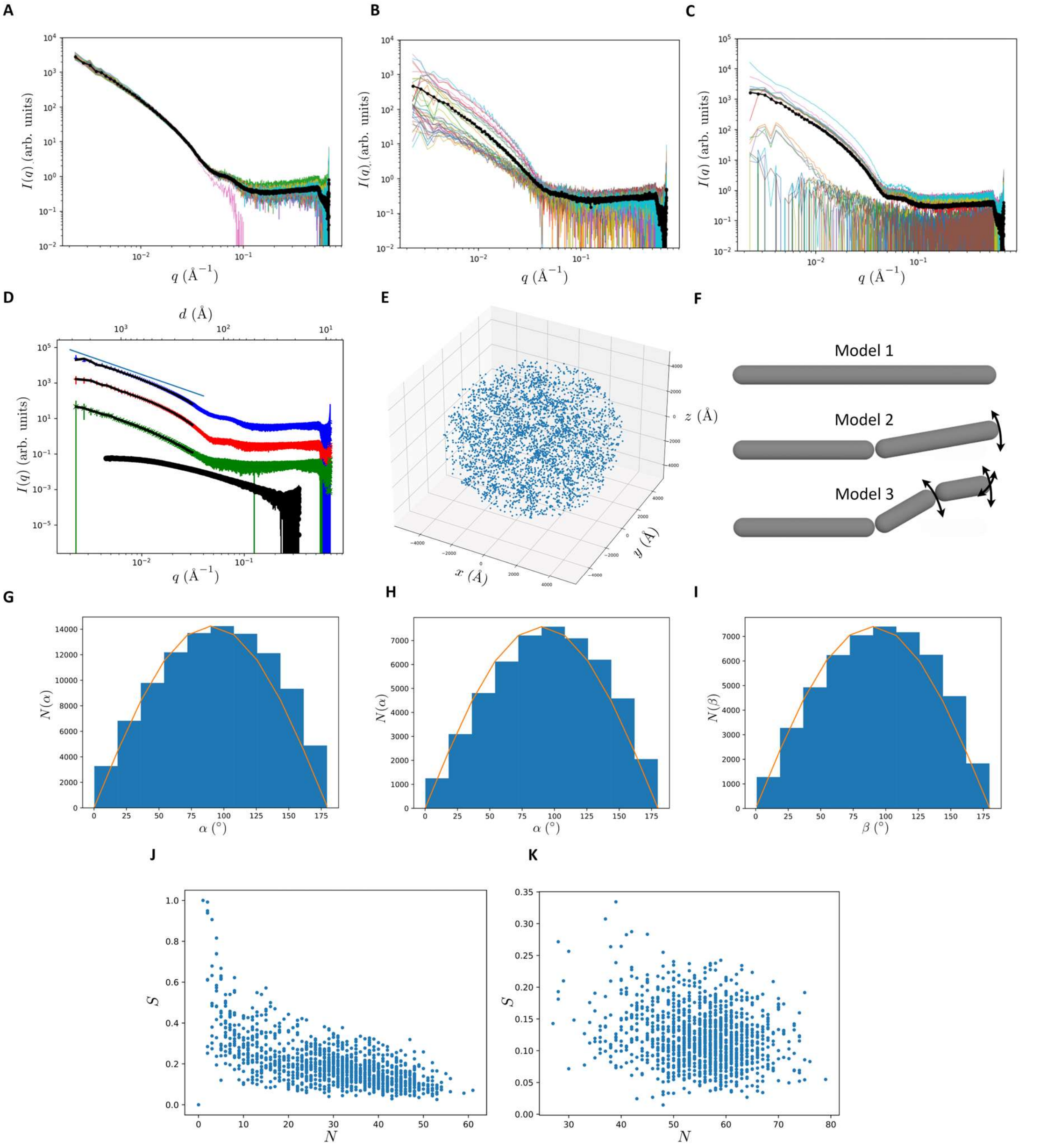

Supplementary Figure 8. SAXS of phase separated Bik1. A-C) SAXS measurements of phase separated Bik1 with subtracted background due to the buffer. Each measurement consists of 40 one-second exposures (thin curves). The variation between exposures is due to the fluctuation of the number of droplets in the X-ray beam, as the sample is continuously pumped through the scattering volume. The average curve, used for further analysis, is shown by the thicker black dash-dotted curve. The measurement shown in panel A is shown in Figure 5A. D) SAXS data of phase-separated Bik1 FL measured in three independent experiments (blue, red, and green curves) and fits (black curves) obtained with 4000 Gaussian blobs in the *q* range (0.002 $Å^{-1}$ - 0.03 $Å^{-1}$) corresponding to distances larger than a fully extended Bik1 dimer. The SAXS data have been shifted with respect to each other in the direction of the Y-axis by one decade for clarity. The form factor obtained from our SEC-SAXS measurements of Bik1 FL (black curve; Figure 2) is shown for comparison. The straight blue line above the SAXS data represents $I(q) \sim q^{-2}$ behavior expected for a fractal network with fractal dimension equal to 2. E) Heterogeneous configuration of Gaussian blob positions in the spherical volume with a diameter of 1 µm resulting from a fit shown in panel D. F) Schematic representations of the three spherocylinder models that were used for data fitting. G, H) Distribution of α angle in the model 2 (G) and model 3 (H). I) Distribution of β angle in model 3. J, K) Local nematic order parameter *S* versus number of model 1 copies, N, in a cubic box. The order parameter is determined locally in cubic boxes with side length 400 Å with respect to the average director determined as the average orientation of the model 1 copies in the box. Plots show 50’000 (J) and 100’000 (K) model 1 copies used to fit the SAXS data. Source data for all panels are provided in the Source Data file.

Supplementary Table 1. R_g and D_{max} values calculated from SEC-SAXS data.

Bik1 variant	Protein concentration [mg/ml][1]	R_g [Å]	D_{max} [Å]
FL	8	98	320
FL	6	91	280
FL	4	88	280
ΔQQFF	8	97	320
ΔQQFF	6	94	294
ΔQQFF	4	94	300
ΔNC	8	83	298
ΔNC	6	80	270
ΔNC	4	79	250

[1]Protein concentrations were measured before the protein was injected into the SEC column.

Supplementary Table 2. SEC-SAXS experimental procedures, data collection, and data analysis.

**Sample details**			
Bik1 variant	FL	ΔQQFF	ΔNC
Organism	*Saccharomyces cerevisiae*		
Source	Expression in *E. coli*		
Sequence	Sequences available in Source Data file		
Extinction coefficient ε (M^{-1} cm^{-1}, at 280 nm)	28420	28670	9970
Molecular weight from chemical composition (kDa)	53.1	52.5	27.7
Molecular weight for the dimer (kDa)	106.2	105.0	55.4
Loading volume/concentration, injection volume, flow rate	50 µl sample at 4 mg/ml was injected at a 0.16 ml/min flow rate	50 µl sample at 6 mg/ml was injected at a 0.16 ml/min flow rate	
SEC column	Shodex KW404-4F		
Solvent composition and source	20 mM Tris-HCl, pH 7.5, supplemented with 500 mM NaCl, 2% glycerol, and 1 mM DTT		
**SAXS data collection parameters**			
Source, instrument and description or reference	Beamline B21 at the Diamond Light Source		
Wavelength (nm)	0.09464		
Sample-to-detector distance (m)	3.7		
q-measurement range ($Å^{-1}$)	0.0045 to 0.34		
Basis for normalization to constant counts	The data were normalized to the intensity of the transmitted beam and radially averaged. The scattering of the solvent-blank was subtracted. Intensity units: cm^{-1} (absolute intensity scaled to water scatter at 0.0163).		
Exposure time, number of exposures	28 successive 3.200 second frames	41 successive 3.200 second frames	37 successive 3.200 second frames
Sample temperature (ºC)	15°C		
**Software employed for SAS data reduction, analysis and interpretation**			
SAS data reduction	ATSAS[3]		
Basic analyses: Guinier, *P*(*r*), scattering particle volume (*e.g.,* Porod volume V_P or volume of correlation V_c)	ATSAS[3]		
Shape/bead modelling	DAMMIF[4], DAMMAVER[5]		
Molecular graphics	PyMOL Molecular Graphics System, Version 2.4.1 Schrödinger, LLC		
**Structural parameters**			
Bik1 variant, *P*(*r*) analysis	FL	ΔQQFF	ΔNC
R_g (Å)	88	94	80
d_{max} (Å)	280	294	270
V_{Porod} (nm^3)	467	632	282
Experimental molecular weight (kDa)	120	124	65
**Ab initio calculation of molecular envelopes**			
Bik1 variant	FL	ΔQQFF	ΔNC
q-range for fitting ($Å^{-1}$)	0.0062 to 0.0958		
Symmetry/anisotropy assumptions	P1		
χ^2 value	0.965	1.029	1.031
Model volume ($Å^3$)	492545	507020	385184
Model resolution (Å)	61 ± 4	65 ± 5	43 ± 3
**Data and model deposition IDs**			
Bik1 variant	FL	ΔQQFF	ΔNC
Deposition ID	SASDUT6	SASDUV6	SASDUU6

Supplementary Table 3. Targeted Bik1 crosslinked peptides measured in this study.

Peptide_ID	Pos_ID
EAQLTIEKLQR-EAQLTIEKLQR-a8-b8	Bik1:207-207:Bik1
EKEIDHLK-KQLHYKR-a2-b6	Bik1:216-245:Bik1
ELFLTKPQDSSSEEVASLTK-ELFLTKPQDSSSEEVASLTK-a6-b6	Bik1:359-359:Bik1
ELFLTKPQDSSSEEVASLTK-KQLHYK-a6-b1	Bik1:211-359:Bik1
ELFLTKPQDSSSEEVASLTK-YQRKIGCFIQIPNLGR-a6-b4	Bik1:7-359:Bik1
ELFLTKPQDSSSEEVASLTKKLEEANEK-K20-156_monolink	Bik1:373
EQLKMHNDSLSK-EQLKMHNDSLSK-a4-b4	Bik1:338-338:Bik1
EQLKMHNDSLSK-KQLHYKR-a4-b6	Bik1:216-338:Bik1
GQLKYVGPVDTK-KLEEANEK-a4-b1	Bik1:23-374:Bik1
KLEEANEK-KLEEANEK-a1-b1	Bik1:374-374:Bik1
KLEEANEKIK-KQLEQLR-a8-b1	Bik1:311-381:Bik1
KQLEQLR-KQLEQLR-a1-b1	Bik1:311-311:Bik1
MHNDSLSKEYQNLSK-EKEIDHLK-a8-b2	Bik1:245-346:Bik1
MHNDSLSKEYQNLSK-KQLHYK-a8-b1	Bik1:211-346:Bik1
MHNDSLSKEYQNLSK-QQAKQK-a8-b4	Bik1:263-346:Bik1
NLSHNQPTGANEDVELLKKQLEQLR-K18-156_monolink	Bik1:310
VASLIEKASISQTSR-EQLKMHNDSLSK-a7-b4	Bik1:79-338:Bik1

Supplementary Table 4. Recombinant proteins and primers used in this study.

**Reagent or Resource**	**Source**	**Identifier**
**Recombinant proteins**		
PSPCm2-6xHis-PreSc-Bik1-1-440	This study	Bik1 FL
PSPCm2-6xHis-PreSc-Bik1-182-440	This study	Bik1 ΔN
PSPCm2-6xHis-PreSc-Bik1-1-436	This study	Bik1 ΔQQFF
PSPCm2-6xHis-PreSc-Bik1-182-396	This study	Bik1 ΔNC
PSPCm2-6xHis-PreSc-mNG-Bik1-1-440	[6]	mNG-Bik1 FL
**Primers**		
Bik1FL-F	This study	CTCTTTCAGGGACCCGATAGATATCAAAGAAAGATAGGATGT
Bik1FL-R	This study	TTGCTAAGTGAGCTCTGTCAATTATCACTAGAAGAACTGCTGGTTGTC
Bik1 ΔN-F	This study	CTCTTTCAGGGACCCAATGGCCACATGAATGACCTCAA
Bik1 ΔNC-R	This study	TTGCTAAGTGAGCTCTGTCAATTATCACGATTCCACGGCTGTTTGTGC
Bik1 ΔQQFF-R	This study	TTGCTAAGTGAGCTCTGTCAATTATCAGTTGTCAGGATTGTGATG

References

1. Jumper, J. *et al.* Highly accurate protein structure prediction with AlphaFold. *Nature* **596**, 583–589 (2021).

2. Ludwiczak, J., Winski, A., Szczepaniak, K., Alva, V. & Dunin-Horkawicz, S. DeepCoil—a fast and accurate prediction of coiled-coil domains in protein sequences. *Bioinformatics* **35**, 2790–2795 (2019).

3. Manalastas-Cantos, K. *et al.* ATSAS 3.0 : expanded functionality and new tools for small-angle scattering data analysis. *J. Appl. Crystallogr.* **54**, 343–355 (2021).

4. Franke, D. & Svergun, D. I. DAMMIF , a program for rapid ab-initio shape determination in small-angle scattering. *J. Appl. Crystallogr.* **42**, 342–346 (2009).

5. Volkov, V. V. & Svergun, D. I. Uniqueness of ab initio shape determination in small-angle scattering. *J. Appl. Crystallogr.* **36**, 860–864 (2003).

6. Meier, S. M. *et al.* Multivalency ensures persistence of a +TIP body at specialized microtubule ends. *Nat. Cell Biol.* **25**, 56–67 (2023).
