# Supplementary material for: Phase separation of a microtubule plus-end tracking protein into a fluid fractal network
Source: Nat Commun. 2025 Jan 30;16:1165. doi: 10.1038/s41467-025-56468-8 (PMC11782662; doi:10.1038/s41467-025-56468-8)
Supplement: Supplementary file 2 — Description of Additional Supplementary Files [file 41467_2025_56468_MOESM2_ESM.pdf]

Description of Additional Supplementary Files

File Name: Supplementary Movie 1
Description: Movie of a “flight” through the Bik1 fractal droplet structure resulting from the fit using 50'000 Bik1 dimers represented by three spherocylinders (model 3; see Figure 5). Densely and loosely packed protein regions are crossed during the flight. A black background is visible in parts of the movie when the flight path gets close to the surface of the spherical volume containing all Bik1 dimer models.

File Name: Supplementary Data 1
Description: Identification of DSS XL peptides in Bik1 variants and different conditions and identification of PDH XL peptides in Bik1 at different conditions.

File Name: Supplementary Data 2
Description: Effect of DSS concentration and fragmentation on the identification of XL peptides.

File Name: Supplementary Data 3
Description: Quantification of DSS XL peptides in Bik1 variants and different conditions.
