# Supplementary material for: Phase separation of a microtubule plus-end tracking protein into a fluid fractal network
Source: Nat Commun. 2025 Jan 30;16:1165. doi: 10.1038/s41467-025-56468-8 (PMC11782662; doi:10.1038/s41467-025-56468-8)
Supplement: Supplementary file 7 — Reporting Summary [file 41467_2025_56468_MOESM7_ESM.pdf]

nature portfolio

Corresponding author(s): Michel Steinmetz

Last updated by author(s): Nov 8, 2024

Reporting Summary

Nature Portfolio wishes to improve the reproducibility of the work that we publish. This form provides structure for consistency and transparency in reporting. For further information on Nature Portfolio policies, see our Editorial Policies and the Editorial Policy Checklist.

Statistics

For all statistical analyses, confirm that the following items are present in the figure legend, table legend, main text, or Methods section.

n/a	Confirmed	
☐	☒	The exact sample size (*n*) for each experimental group/condition, given as a discrete number and unit of measurement
☐	☒	A statement on whether measurements were taken from distinct samples or whether the same sample was measured repeatedly
☐	☒	The statistical test(s) used AND whether they are one- or two-sided<br>*Only common tests should be described solely by name; describe more complex techniques in the Methods section.*
☒	☐	A description of all covariates tested
☒	☐	A description of any assumptions or corrections, such as tests of normality and adjustment for multiple comparisons
☐	☒	A full description of the statistical parameters including central tendency (e.g. means) or other basic estimates (e.g. regression coefficient) AND variation (e.g. standard deviation) or associated estimates of uncertainty (e.g. confidence intervals)
☐	☒	For null hypothesis testing, the test statistic (e.g. *F*, *t*, *r*) with confidence intervals, effect sizes, degrees of freedom and *P* value noted<br>*Give P values as exact values whenever suitable.*
☒	☐	For Bayesian analysis, information on the choice of priors and Markov chain Monte Carlo settings
☒	☐	For hierarchical and complex designs, identification of the appropriate level for tests and full reporting of outcomes
☒	☐	Estimates of effect sizes (e.g. Cohen's *d*, Pearson's *r*), indicating how they were calculated

Our web collection on statistics for biologists contains articles on many of the points above.

Software and code

Policy information about availability of computer code

Data collection	Mass Spectrometry data was acquired on Thermo Fischer Scientific mass Spectrometer operated with XCalibur 4.1 Software.<br>CD measurements were done using Chirascan-Plus spectrophotometer (Applied Photophysics Ltd.).<br>Fluorescently tagged Bik1 (mNeonGreen) was imaged using an inverted confocal microscope (Nikon Ti2 Eclipse with Yokogawa CSU-W1 spinning disc). For the SEC-SAXS measurements, beamline control was provided by the Generic Data Acquisition, GDA, software developed at Diamond Light Source (https://www.opengda.org/). SAXS data for the phase-separated droplets of Bik1 were collected using “cSAXS_base_package” as described on https://www.psi.ch/en/sls/csaxs/software.
Data analysis	Crosslink data was analyzed using xQuest v2.1.138.<br>SEC-SAXS data was analyzied using ATSAS 3.2.1 package with DAMMIF, DAMAVER, SASRES;<br>Protein visualizations were prepared using PyMOL Molecular Graphics System, Version 2.4.1 Schrödinger.<br>Intensities inside fluorescently tagged droplets were analyzed using MATLAB.<br>The data analysis procedures underlying this article are described in the main text and the Supplementary Information.

For manuscripts utilizing custom algorithms or software that are central to the research but not yet described in published literature, software must be made available to editors and reviewers. We strongly encourage code deposition in a community repository (e.g. GitHub). See the Nature Portfolio guidelines for submitting code & software for further information.

Data

Policy information about availability of data

All manuscripts must include a data availability statement. This statement should provide the following information, where applicable:

- Accession codes, unique identifiers, or web links for publicly available datasets
- A description of any restrictions on data availability
- For clinical datasets or third party data, please ensure that the statement adheres to our policy

Custom R script code and MS files are available in the PRIDE archive, a public data repository for MS proteomics data (https://www.ebi.ac.uk/pride/), with the following IDs: PXD050928, PXD050929, PXD050930, PXD050931. The SEC-SAXS datasets can be found in the SASDB archive, Small Angle Scattering Biological Data Bank (https://www.sasbdb.org/) with the following accession codes: SASDUT6, SASDUU6, SASDUV6. The SAXS datasets collected for the phase-separated droplets of Bik1 are deposited in the Zenodo (https://zenodo.org/) repository, with the following DOI: 10.5281/zenodo.10944717. The code used to fit the SAXS curves of phase separated droplets of Bik1 can be found in the Zenodo repository with the following DOI: 10.5281/zenodo.13822678.

Research involving human participants, their data, or biological material

Policy information about studies with human participants or human data. See also policy information about sex, gender (identity/presentation), and sexual orientation and race, ethnicity and racism.

Reporting on sex and gender	n/a
Reporting on race, ethnicity, or other socially relevant groupings	n/a
Population characteristics	n/a
Recruitment	n/a
Ethics oversight	n/a

Note that full information on the approval of the study protocol must also be provided in the manuscript.

Field-specific reporting

Please select the one below that is the best fit for your research. If you are not sure, read the appropriate sections before making your selection.

[x] Life sciences [ ] Behavioural & social sciences [ ] Ecological, evolutionary & environmental sciences

For a reference copy of the document with all sections, see nature.com/documents/nr-reporting-summary-flat.pdf

Life sciences study design

All studies must disclose on these points even when the disclosure is negative.

Sample size	Statistical methods were not used to predetermine sample size. For quantitative XL-MS experiments, measurements of n = 3 biologically independent samples (except for the pellet conditions where n=1) were performed in order to allow statistical testing.
Data exclusions	No data were excluded.
Replication	All attempts at replication were successful.
Randomization	No randomization was done in this study.
Blinding	No blinding was done in this study.

Reporting for specific materials, systems and methods

We require information from authors about some types of materials, experimental systems and methods used in many studies. Here, indicate whether each material, system or method listed is relevant to your study. If you are not sure if a list item applies to your research, read the appropriate section before selecting a response.

Materials & experimental systems

n/a	Involved in the study	
☒	☐	Antibodies
☒	☐	Eukaryotic cell lines
☒	☐	Palaeontology and archaeology
☒	☐	Animals and other organisms
☒	☐	Clinical data
☒	☐	Dual use research of concern
☒	☐	Plants

Methods

n/a	Involved in the study	
☒	☐	ChIP-seq
☒	☐	Flow cytometry
☒	☐	MRI-based neuroimaging

Plants

Seed stocks	n/a
Novel plant genotypes	n/a
Authentication	n/a
